# ARA290, a non-erythropoietic EPO derivative, attenuates renal ischemia/reperfusion injury

**DOI:** 10.1186/1479-5876-11-9

**Published:** 2013-01-09

**Authors:** Willem G van Rijt, Gertrude J Nieuwenhuijs-Moeke, Harry van Goor, Bente Jespersen, Petra J Ottens, Rutger J Ploeg, Henri GD Leuvenink

**Affiliations:** 1 Department of Surgery, University of Groningen, University Medical Center Groningen, Groningen, the Netherlands; 2 Department of Pathology and Medical Biology, University of Groningen, University Medical Center Groningen, Groningen, the Netherlands; 3 Department of Anaesthesiology, University of Groningen, University Medical Center Groningen, Groningen, the Netherlands; 4 Department of Renal Medicine, Aarhus University Hospital, Skejby, Denmark; 5 Nuffield Department of Surgical Sciences, University of Oxford, Oxford, UK

**Keywords:** ARA290, Pyroglutamate helix B-surface peptide, Erythropoietin, Ischemia/reperfusion injury, Kidney transplantation

## Abstract

**Background:**

In contrast with various pre-clinical studies, recent clinical trials suggest that high dose erythropoietin (EPO) treatment following kidney transplantation does not improve short-term outcome and that it even increases the risk of thrombotic events. ARA290 is a non-erythropoietic EPO derivative and does not increase the risk of cardiovascular events, but potentially has cytoprotective capacities in prevention of renal ischemia/reperfusion injury.

**Methods:**

Eight female Dutch Landrace pigs were exposed to unilateral renal ischemia for 45 minutes with simultaneous cannulation of the ureter of the ischemic kidney. ARA290 or saline was administered by an intravenous injection at 0, 2, 4 and 6 hours post-reperfusion. The animals were sacrificed seven days post-reperfusion.

**Results:**

ARA290 increased glomerular filtration rate during the observation period of seven days. Furthermore, ARA290 tended to reduce MCP-1 and IL-6 expression 15 minutes post-reperfusion. Seven days post-reperfusion ARA290 reduced interstitial fibrosis.

**Conclusions:**

The improvement in renal function following renal ischemia/reperfusion and reduced structural damage observed in this study by ARA290 warrants further investigation towards clinical application.

## Background

Despite many achievements in transplantation the persistent shortage of deceased donors remains a major problem. This shortage resulted in an evident increase in living donation, but also forced transplant teams to accept more kidneys from marginal deceased brain death (DBD) donors and deceased cardiac death (DCD) donors. In 2011, 50% of the deceased donor kidneys in the Netherlands were retrieved from DCD donors while in 1996 this was only 6% [1,2].

A donor kidney is by definition exposed to a period of ischemia which lasts until the kidney is re-connected to the circulation of the recipient. This period of ischemia can be divided into three phases: primary warm ischemia, cold storage and secondary warm ischemia. Primary warm ischemia is the time between end of cardiovascular circulation and start of cold storage. Cold storage starts when the donor organ is flushed with 4°C preservation solution. Secondary warm ischemia is the time between end of cold storage and reperfusion of the donor organ in the recipient. The whole ischemic process results in a cascade causing renal damage and is associated with the phenomenon of ischemia/reperfusion (I/R) injury. In DCD donation, the primary period of warm ischemia is variable and explicitly extended compared to the controlled circumstances in DBD donation. This results in a significant increase in the risk of delayed graft function (DGF) and primary non function (PNF). Death censored graft survival at 15 years of DCD kidneys is significantly reduced compared to DBD kidneys, 46% and 60%, respectively. Excluding primary non-functioning kidneys equals long-term graft survival of DCD and DBD kidneys [3]. Thus, short-term function of DCD kidneys appears to be crucial for long-term survival. The major part of I/R injury manifests itself during the reperfusion phase. Therefore, cytoprotective treatment early on during the reperfusion phase could key to enhance immediate function and improve long-term graft survival of DCD kidneys.

Erythropoietin (EPO) is a potential cytoprotective glycoprotein. In renal I/R models it has been shown that administration of EPO before reperfusion [4] as well as after reperfusion [5] can be cytoprotective. Protective EPO treatment has pronounced anti-inflammatory and anti-apoptotic capacities [5,6]. Enhanced activity of endothelial nitric oxide synthase (eNOS) is presumably responsible for the immediate effect of EPO on renal function [7,8]. However, the exact pathway of cytoprotection by EPO is not yet fully elucidated. The proposed cytoprotective mechanism is binding of EPO to a heteromeric EPOR_2_-βcR_2_ receptor complex consisting of two EPO receptors and two beta common receptors (EPOR_2_-βcR_2_) [9]. The binding affinity of the classic, erythropoietic complex of two EPO receptors for EPO is distinctly larger than the binding affinity of the protective EPOR_2_-βcR_2_ complex [10]. Thus, to induce local tissue protection, considerably higher systemic doses of EPO are required than the normal therapeutic doses used for stimulation of erythropoiesis [10].

Renal I/R models have been widely used to mimic warm ischemic injury following DCD donation. Based on pre-clinical renal I/R experiments, activation of the protective EPO pathway could improve quality of DCD kidney [4-6]. Recently, the protective effect of high dose EPO treatment in deceased kidney transplantation has been evaluated in four clinical studies. None of these studies was able to demonstrate a reduction of DGF or PNF [11-14]. High dose EPO treatment even resulted in an increased risk of thrombotic events in the first year after transplantation (Epoetin 24.4% vs. placebo 6.4%) [13]. However, the EPO doses were relatively low to induce cytoprotection compared to used doses in most animal models [4-6]. Therefore it is questionable whether protective, systemic EPO levels were obtained in these clinical trials, although the risk of cardiovascular adverse events was already increased.

To prevent these cardiovascular adverse effects, EPO derivatives have been developed which only activate the protective EPOR_2_-βcR_2_ complex and do not stimulate erythropoiesis. ARA290 is the newest generation EPO analogue. It is a small synthetic peptide, which selectively binds to the EPOR_2_-βcR_2_ complex. It has already been shown that ARA290, also known as pyroglutamate helix B-surface peptide (pHBSP), is not erythropoietic [15]. Patel et al showed that administration of ARA290 or EPO after reperfusion improves renal function in a rodent model of renal I/R. Mechanistically, ARA290 and EPO increase the phosphorylation of survival pathway Akt. Activation of pro-inflammatory glycogen synthase kinase-3β and nuclear factor-κβ were inhibited by ARA290 and EPO. Furthermore, ARA290 and EPO were able to increase phosphorylation of eNOS [16]. The effect of ARA290 on these pathways could explain both the anti-inflammatory capacities and the effect on renal function.

Diminution of I/R injury in rodents showed the potential of ARA290 as cytoprotective agent following transplantation. We hypothesized that post-reperfusion administered ARA290 protects against renal I/R injury. For that purpose, ARA290 was tested in a porcine renal I/R model.

## Materials and methods

### Animals

Eight Dutch Landrace pigs (50-70 kg.) were used. They were housed individually with free access to water. The pigs were fasted overnight before surgery.

The animal experiments were approved by the animal ethics committee of the university Groningen (DEC-RuG, 4762, Groningen, the Netherlands) and were performed according to international and Dutch guidelines of animal research. The experiment was designed according to the principle of replacement by alternative methods, reduction of the number of animals and refinement of experiments as EU policy as laid out in Towards Responsible Animal Research (EMBO R 4, 104-107). Based on the assumed effect size a minimal group size of four was calculated.

### ARA290

ARA290 (ARAIM Pharmaceuticals, Ossining, USA) is a small synthetic peptide consisting of eleven amino acids. It has been derived from the binding site of EPO to the protective EPOR_2_-βcR_2_ complex and it does not bind to the classical EPOR_2_ complex. The plasma half-life is approximately 2 minutes.

### I/R model

In this study we used a unilateral I/R model without contralateral nephrectomy to reduce animal discomfort as a refinement. The pigs were randomized either into the vehicle treated control group or the ARA290 group. Four animals were included in each group. Saline was used as vehicle treatment. ARA290 (10 nmol/kg) or saline was administered intravenously. Animals were treated at 0, 2, 4 and 6 hours post-reperfusion.

Prior to the procedure ketamine, xylazine and atropine were administered intramuscularly to reduce anxiety. General anaesthesia was induced by intravenous administration of midazolam and maintained with isoflurane 2,5%. Intubation and surgical conditions were optimized using pancuronium and analgesia was performed with fentanyl. During the procedure the animals were mechanically ventilated.

The jugular vein and the carotid artery were cannulated and surgery was started by a medial laparotomy. The left renal artery and renal vein were dissected and prior to clamping heparin (70 IU/kg) was injected intravascularly. Prior to ischemia a blood sample as well as a needle biopsy were taken. Baseline renal cortical perfusion was checked by Laser Doppler (t=-45). Renal ischemia for 45 minutes was induced by clamping both the renal artery and renal vein. During ischemia the ureter of the ischemic kidney was cannulated and subsequently tunnelled to the dorsal site of the animal. The cannula ended in a small backpack and it was connected to a swivel system. After 43 minutes of ischemia a blood sample and biopsy of the ischemic kidney were taken (t=-2). At t=0 blood flow was restored by removal of the clamps. At t=15 the last needle biopsy was taken as well as a blood sample after which the abdomen was closed. Animals were allowed to wake up and housed in adjacent metabolic cages in a temperature controlled environment

During the seven day follow-up, daily blood samples were taken via the cannulated jugular vein enabling stress free blood sampling. Urine was collected continuously via swivel system connected to the ischemic kidney.

After seven days animals were anesthetized and the kidneys were exposed. Before removal of both the ischemic and the contralateral kidneys, they were flushed via the aorta using Ringer’s Lactate at 4°C. Subsequently all animals were sacrificed by administration of lethal dose of pentobarbital.

### Samples

Blood and urine samples were stored at -80°C. A cortical sample of the kidney was snap frozen in liquid N_2_ and stored at -80°C for qRT-PCR analyses. For immunohistochemistry and morphology a cortical sample was fixated in 4% formalin and subsequently embedded in paraffin.

### Glomerular filtration rate

Primary endpoint of the study was renal function defined as glomerular filtration rate (GFR). In our model plasma creatinine levels cannot be used to monitor renal function as the healthy, contralateral kidney will compensate the loss of renal function of the I/R kidney. To be able to measure renal function of the I/R kidney, we cannulated the ureter of this kidney. In this way we were able to monitor urinary output of the I/R kidney continuously. This technique diminishes animal discomfort which justifies the more complex determination of renal function. The GFR of the I/R kidney was calculated using plasma creatinine levels and urinary creatinine excretion of the I/R kidney. The GFR was calculated using the following formula: GFR = urinary creatinine level (mM) * urine volume (ml)/plasma creatinine level (mM) * time (minutes).

### Real-time reverse transcription polymerase chain reaction (qRT-PCR)

RNA was extracted from snap frozen tissue using Trizol reagent according to the manufacturer’s instructions (Invitrogen, Breda, the Netherlands). Total RNA was treated with DNAse I to remove genomic DNA contamination (Invitrogen, Breda, the Netherlands). The integrity of total RNA was analysed by gel electrophoresis. cDNA synthesis was performed from 1-μg total RNA using M-MLV (Moloney murine leukaemia virus) Reverse Transcriptase and oligo-dT primers (Invitrogen, Breda, The Netherlands).

Primer sets were designed using Primer Express 2.0 software (Applied Biosystems, Foster City, CA). Amplification and detection were performed with the ABI Prism 7900-HT Sequence Detection System (Applied Biosystems) using emission from SYBR green master mix (Applied Biosystems). The PCR reactions were performed in triplicate. After an initial activation step at 50°C for 2 min and a hot start at 95°C for 10 min, PCR cycles consisted of 40 cycles at 95°C for 15 sec and 60°C for 60 sec. Dissociation curve analysis were performed for each reaction to ensure amplification of specific products.

Gene expression of TNF-α, IL-6, MCP-1, α-SMA, TGF-β and β-actin (housekeeping gene) were determined. Primers are shown in Table 1. Gene expression was normalized with the mean of β-actin mRNA content and calculated relative to controls or contralateral kidneys. Results were finally expressed as 2–ΔCT (CT threshold cycle), which is an index of the relative amount of mRNA expression in each tissue.

**Table 1:** qRT-PCR primers

**Primers:**	**Forward:**	**Reverse:**	**Amplicon length (bp):**
β-actin	TCTGCGCAAGTTAGGTTTTGTC	CGTCCACCGCAAATGCTT	78
TNF-α	GGCTGCCTTGGTTCAGATGT	CAGGTGGGAGCAACCTACAGTT	63
IL-6	AGACAAAGCCACCACCCCTAA	CTCGTTCTGTGACTGCAGCTTATC	69
MCP-1	ACTTGGGCACATTGCTTTCCT	TTTTGTGTTCACCATCCTTGCA	84
α-SMA	ACGAAGCCCAAAGCAAAAGA	GTTGGTGATGATGCCGTGTTC	67
TGF- β	GGGAGGGTGTTCATGGTAGGA	AGCTCACCCCAAATTCATCTTC	66

### Immunohistochemistry

In paraffin embedded kidney samples were cut into 3-μm-thick sections. The morphology was evaluated by Periodic Acid-Schiff (PAS) staining. Immunohistochemical staining for α-SMA (pre-fibrotic changes) was performed.

Deparaffinised sections were subjected to antigen retrieval. The sections for α-SMA staining were incubated for one night in 0.1M Tris/HCl buffer (pH 9.0).

Next, endogenous peroxidase was blocked using 0,3% H2O2 for 30 minutes. The incubation time of the α-SMA antibody (1:10000, Clone 1A4, Sigma) was 1 hour. For the α-SMA staining we used a secondary peroxidase-conjugated goat-anti-mouse antibody (1:100, DAKO, Glostrup, Denmark) and a tertiary peroxidase-conjugated rabbit-anti-goat antibody (1:100, DAKO, Glostrup, Denmark). The incubation time of the secondary and tertiary antibodies was 30 minutes. Then the peroxidase activity was visualized by 3,3-diaminobenzidine tetrachloride (DAB) incubation for 10 minutes.

Subsequently, the sections were scanned using APERIO scanscope (Aperio, Vista, United States). The expression of the immunohistochemical staining of each section was quantified using APERIO image scope software.

### NOx determination

Total nitrite and nitrate levels (NOx) in blood and urine were used as a marker of nitric oxide synthase activity. As nitrite oxidizes rapidly to nitrate, we used the enzyme nitrate reductase to convert nitrate into nitrite. Next the nitrite levels were determined using the Griess reaction [17]. NOx clearance was calculated using the following formula: NOx clearance = urine flow (ml/min) * urinary NOx (μmol/ml)/plasma NOx (μmol/ml).

### Statistical analyses

All data are presented as mean±standard error of the mean (SEM). To analyse the data the two-way ANOVA or the Mann-Whitney U test was used depending on type of data. p<0.05 was considered significant.

## Results

### ARA290 improved renal function

**Figure 1:**
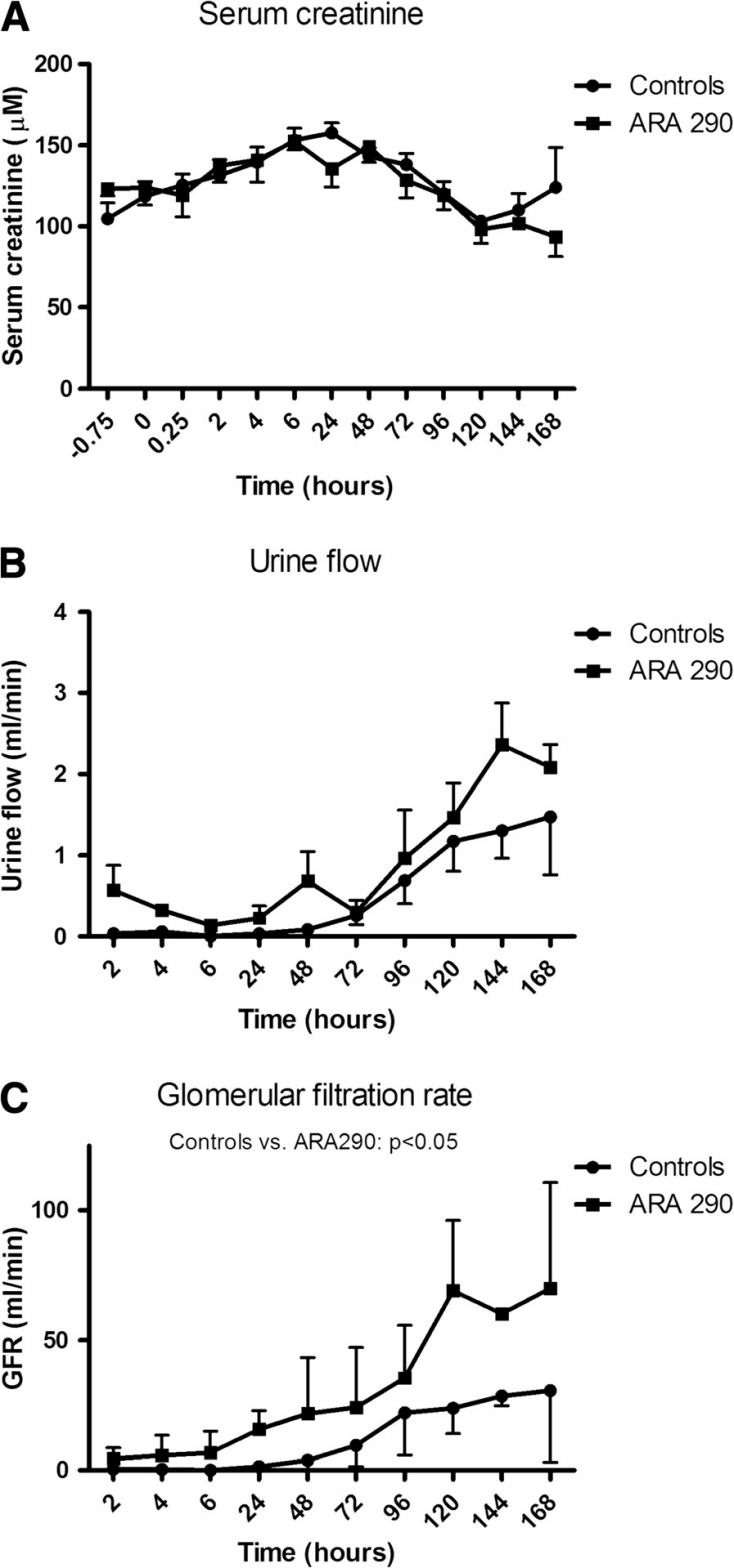
**Effect of ARA290 on clinical markers of renal function.** No differences were found in plasma creatinine levels (**1A**) or urine flow (**1B**) between vehicle- or ARA290 treated animals. The GFR of ARA290 treated animals was significantly increased in the first seven days post-reperfusion. ARA290 resulted in immediate renal function, while control kidneys started functioning after 48 hours (**1C**, p<0.05).

Plasma creatinine levels of all animals increased in the first 24 hours after reperfusion. After the first day, plasma creatinine levels declined during the remaining 6 days. Plasma creatinine levels and urine flow did not differ between vehicle- and ARA290 treated animals (Figure 1A and B). The glomerular filtration rate (GFR) of the I/R kidney was calculated based on daily plasma creatinine levels and 24-hours urine via the cannulated ischemic kidney. The GFR of the I/R kidney was markedly increased by ARA290 treatment post-reperfusion in the first seven days post-reperfusion (p<0.05, Figure 1C). No differences were found in haemoglobin, haematocrit, urea or aspartate transaminase plasma levels. No cardiovascular adverse events were observed in vehicle- or ARA290 treated animals.

### The anti-inflammatory effect of ARA290

**Figure 2:**
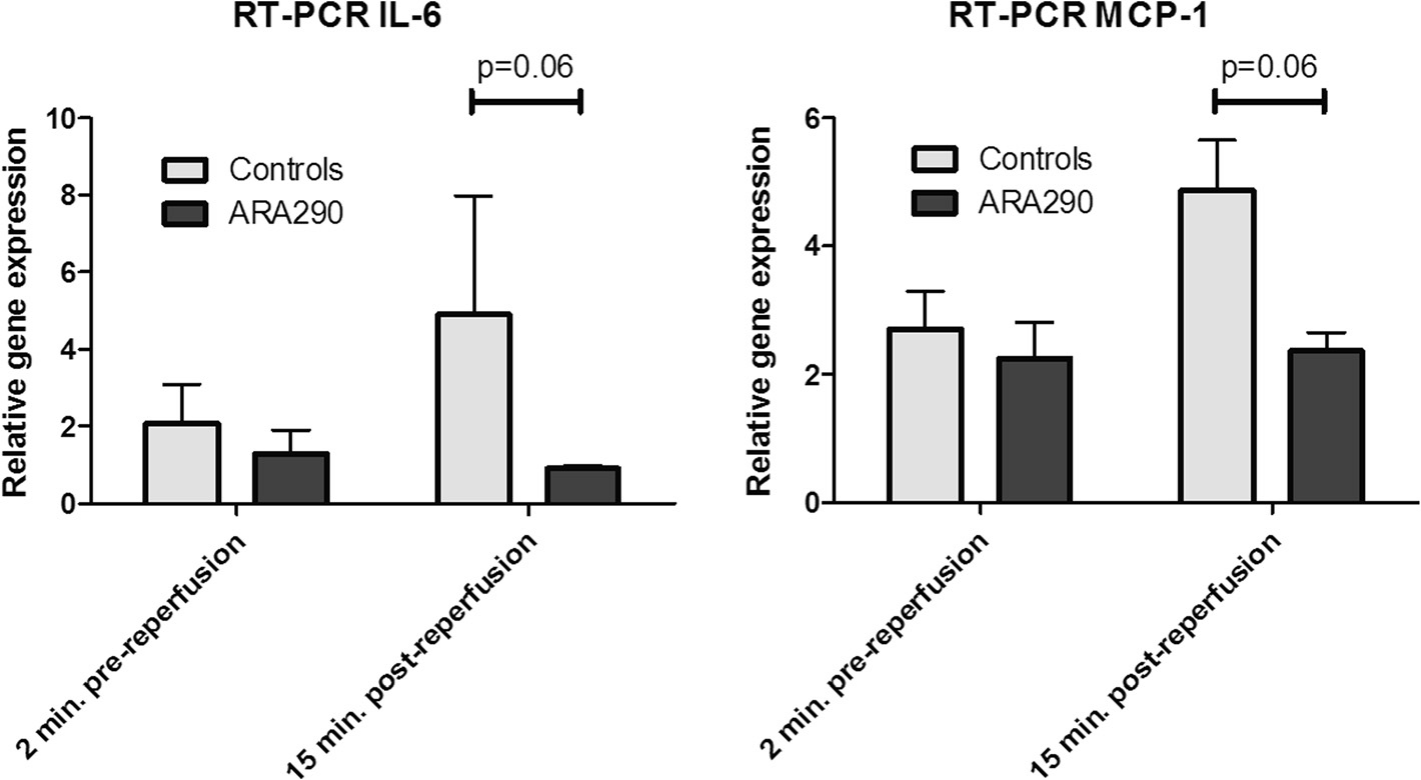
**Effect of ARA290 on acute inflammation.** Warm ischemia doubled mRNA expression of IL-6 and MCP-1 at 2 minutes prior to reperfusion. ARA290 tends to reduce mRNA expression of IL-6 and MCP-1 relative to baseline only 15 minutes after the first administration of ARA290, indicative of its direct anti-inflammatory effect (p=0.06).

Two minutes prior to reperfusion, after 43 minutes of ischemia, interleukin-6 (IL-6) and monocyte chemotactic protein-1 (MCP-1) mRNA expression doubled. At this time point ARA290 treatment has not yet been started and there are no differences in mRNA expression of IL-6 or MCP-1 (Figure 2). ARA290 tended to reduce an early increase of IL-6 and MCP-1 mRNA expression at 15 minutes post-reperfusion relative to the baseline (p=0.06, Figure 2). In contrast to IL-6 and MCP-1, ARA290 did not influence tumour necrosis factor- α (TNF-α) mRNA expression at 15 minutes post-reperfusion. Seven days post-reperfusion no differences in inflammation were found.

### ARA290 reduced structural damage

**Figure 3:**
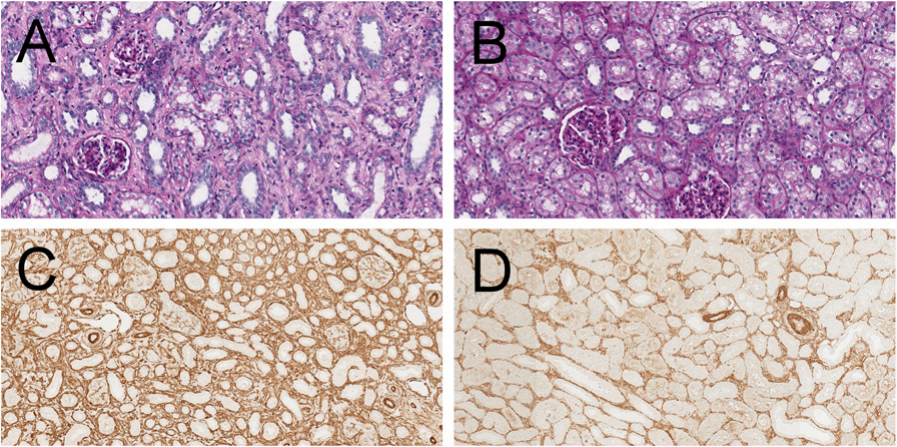
**Effect of ARA290 on structural damage.** The morphology of ischemic kidneys at seven days post-reperfusion is shown by a PAS staining in representative sections of control group (**3A**) and ARA290 treated group (**3B**). ARA290 attenuated morphological injury at seven days post-reperfusion. Immunohistochemical α-SMA staining shows expression at seven days post-reperfusion in the control group (**3C**) and the ARA290 treated group (**3D**).

**Figure 4:**
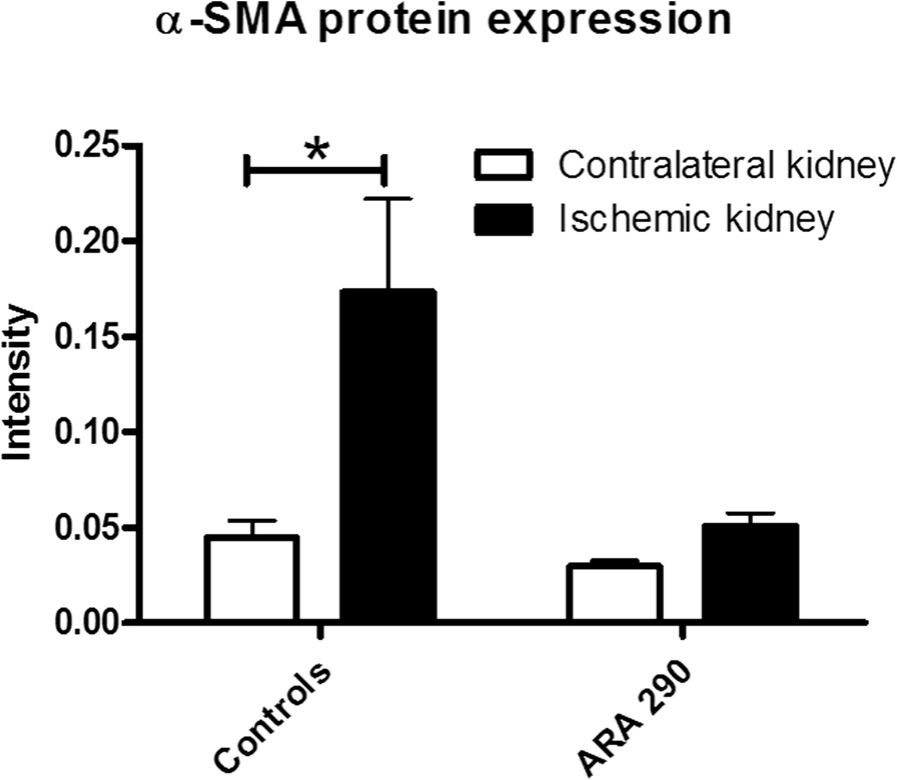
**Effect of ARA290 on interstitial α-SMA expression.** Intensity of the interstitial α-SMA staining has been quantified using APERIO scanscope. ARA290 prevented an increase of interstitial α-SMA expression relative to the contralateral kidneys (4, p<0.05).

**Figure 5:**
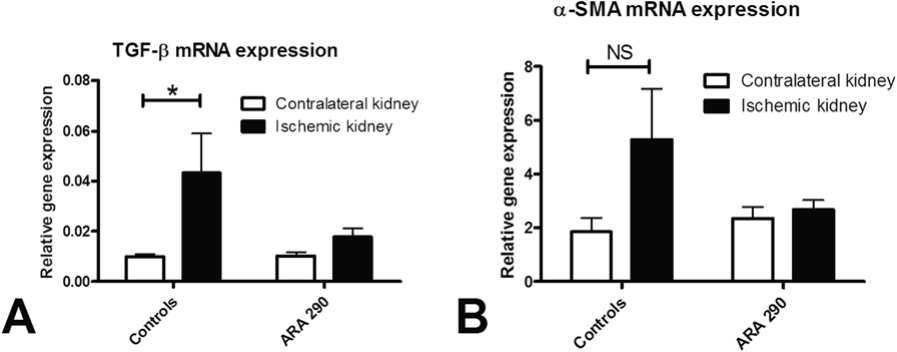
**Effect of ARA290 on mRNA expression of markers of structural damage.** qRT-PCR analyses show TGF-β and α-SMA mRNA expression at seven days post-reperfusion relative to contralateral kidney. ARA290 prevented an increase of TGF-β mRNA expression (p<0.05, **5A**). A similar tendency was observed in α-SMA mRNA expression (**5B**).

In the control group I/R kidneys show morphological interstitial fibrotic injuries by PAS staining. These processes are markedly reduced in ARA290 treated animals (Figure 3A and B). To further investigate these interstitial processes an immunohistochemical staining for α –smooth muscle actin (α-SMA) was performed (Figure 3C and D). Quantification of the intensity showed increased interstitial α-SMA expression in the ischemic kidneys of the controls compared to the contralateral kidneys, indicative of fibrotic changes. ARA290 prevented the interstitial increase of α-SMA expression (p<0.05, Figure 4). In addition to the immunohistochemical staining, α-SMA mRNA and transforming growth factor- β (TGF-β) mRNA expression were measured. ARA290 prevented the increase of TGF-β mRNA expression in the ischemic kidney at seven days post-reperfusion relative to the contralateral kidney. Also an increase in α-SMA mRNA expression tends to be prevented (Figure 5A and B).

### The effect of ARA290 on nitric oxide synthase activity

**Figure 6:**
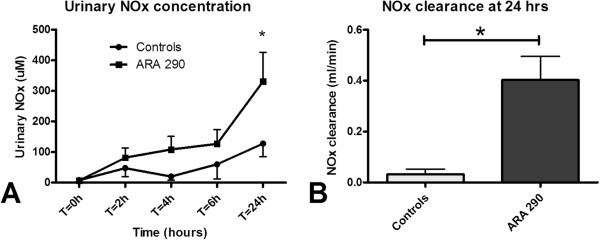
**Effect of ARA290 on urinary NOx concentration and NOx clearance.** ARA290 tended to result in an immediate increase in urinary NOx concentration. At 24 hours post-reperfusion urinary NOx concentration is significantly increased by ARA290 (p<0.05, **6A**). At 24 hours post-reperfusion administered ARA290 increased NOx clearance (**6B**, p<0.05).

ARA290 did not influence total nitrite and nitrate (NOx) plasma levels. However, 24 hours post-reperfusion urinary NOx excretion of ARA290 treated animals was significantly increased compared to the controls (p<0.05, Figure 6A). ARA290 also increased NOx clearance compared to vehicle treatment at 24 hours post-reperfusion (p<0.05, Figure 6B).

## Discussion

The present study shows that ARA290, a synthetic EPO derivative, administrated post-reperfusion is beneficial in renal ischemia reperfusion injury. ARA290 increased GFR in the first seven days post-reperfusion, indicative of improved renal function. Moreover, ARA290 prevented structural damage evidenced by reduced α-SMA and TGF-β expression. ARA290 tends to reduce acute inflammation. Importantly, we did not observe any thrombotic or other cardiovascular adverse effects in ARA290 treated animals. The number of animals was calculated for the primary endpoint and limits any conclusions concerning secondary outcomes. Nevertheless, the effect of ARA290 on the main outcome parameter, renal function, is profound.

A number of studies have shown that EPO and EPO analogues can be renoprotective in renal I/R injury [4-6]. It has already been shown in vitro and in vivo that ARA290 does not bind the erythropoietic EPOR_2_ complex [9,15]. This suggests that the main cytoprotective route of EPO and EPO analogues is mediated by binding to the EPOR_2_-βcR_2_ receptor complex. Unfortunately, the exact distribution of this receptor complex in renal tissue is unclear as there is no antibody for the EPOR_2_-βcR_2_ receptor complex available. Cassis et al. observed tubular staining of both the EPOR and βcR in healthy kidneys. Ischemia resulted in increased tubular expression of the EPOR. Besides also the vessels and glomeruli showed faint EPOR and βcR staining after ischemia [18]. This suggests EPOR and βcR are distributed in both vascular and tubular tissue after renal ischemia/reperfusion injury. The proposed cytoprotective pathway of the EPOR_2_-βcR_2_ receptor complex is the Janus kinase-2 (JAK-2) pathway. In vitro it has been shown that the JAK-2 pathway is required for protection by EPO [19]. Phosphorylation of JAK-2 results in potent anti-inflammatory and anti-apoptotic effects. ARA290 influences downstream pathways of JAK-2, such as activation of pro-survival pathway Akt and inhibition of pro-inflammatory pathways p38 mitogen activated protein kinase, glycogen synthase kinase-3β and nuclear factor-κβ [20-22]. In this study design it was not possible to measure activation of these pathways as tissue samples suitable for western blot analysis were obtained seven days post-reperfusion.

The anti-inflammatory or anti-apoptotic effects do not explain the immediate improvement of renal function. Activation of eNOS is also a downstream result of JAK-2 phosphorylation [7] and most interestingly eNOS is able to induce a direct increase of GFR [23]. Patel et al. showed that ARA290 treatment after renal ischemia increases eNOS phosphorylation in rats. Our study was focussed on the functional effect of ARA290 and in this study design we are not able to measure eNOS phosphorylation. However, we did observe increased urinary NOx clearance. This suggests an increased activity of nitric oxide synthase. Unfortunately, we cannot distinguish between NOx derived from activated eNOS or the inflammatory, inducible NOS. Based on the anti-inflammatory capacities of ARA290 and the increased eNOS phosphorylation shown by Patel et al., the increased NOx clearance is presumably the result of enhanced eNOS activity. The cytoprotective pathway of ARA290 needs further investigation to fully understand this promising way of tissue protection.

## Conclusions

The immediate stimulative effect of ARA290 treatment post-reperfusion makes ARA290 a potential drug to reduce DGF and PNF following renal transplantation. Its effect on structural damage suggests that also graft survival could be positively affected by ARA290 treatment post-transplantation. The advantage of ARA290 when compared to other cytoprotective treatments is the moment of administration. The majority of protective agents or regimen against I/R injury have to be administered prior to- or during ischemia, while ARA290 can be administered post-reperfusion.

This has significant clinical advantages: especially, in DCD transplantation it is ethically and practically not possible to treat the donor. Therefore, cytoprotective treatment of the recipient is necessary to improve short- and long-term outcome of organs retrieved from these donors. Since DCD donors are far more available than DBD donors, improving the quality and function of DCD donor kidneys is an opportunity to reduce the shortage of donor organs.

In conclusion, this study shows the renal protective effect of early administration of ARA290 post-reperfusion. ARA290 lacks the erythropoietic capacities of EPO and does not increase the risk of cardiovascular adverse events. Therefore, ARA290 may translate this promising way of cytoprotection to the transplantation clinic. Further research should focus on the protective mechanism and the long-term effect of ARA290 in models of renal transplantation. Based on the results of this study, ARA290 is a promising drug to prevent I/R injury and to improve renal function rapidly following renal transplantation.

## Abbreviations

α-SMA: α-smooth muscle actin; βcR: β common receptor; DBD: Donation after brain death; DCD: Donation after cardiac death; DGF: Delayed graft function; eNOS: Endothelial nitric oxide synthase; EPO: Erythropoietin; EPOR: Erythropoietin receptor; GFR: Glomerular filtration rate; IL-6: Interleukin-6; I/R: Ischemia/reperfusion; JAK-2: Janus kinase-2; MCP-1: Monocyte chemotactic protein-1; NOx: Nitrite + nitrate; pHBSP: Pyroglutamate helix B-surface peptide; PNF: Primary non function; qRT-PCR: Real-time reverse transcription polymerase chain reaction; TGF- β: Transforming growth factor-β; TNF-α: Tumour necrosis factor-α.

## Competing interest

All authors declare no conflict of interests. ARAIM pharmaceuticals (Ossining, USA) provided an unrestricted grant for this study.

## Authors’ contributions

WGR participated in research design, performance of the study, data analysis and writing of the paper. GJN participated in data analysis and revising of the paper. HG participated in research design, data analysis and revising of the paper. BJ participated in revising of the paper. PJO participated in performance of the research. RJP participated in research design and revising of the paper. HGDL participated in research design, performance of the research, data analysis and revising of the paper. All authors read and approved the final manuscript.
